# The relationship between superior vena caval and mixed venous saturations after cardiac surgery

**DOI:** 10.1186/1749-8090-8-S1-P62

**Published:** 2013-09-11

**Authors:** H Gasparovic, R Gabelica, Z Ostojic, T Kopjar, M Petricevic, B Biocina

**Affiliations:** 1 Department of Cardiac Surgery University Hospital Center Zagreb, Zagreb, Croatia; 2 Department of Anesthesiology and Critical Care Medicine, University Hospital Zagreb, Zagreb, Croatia; 3 The University of Zagreb, School of Medicine, Zagreb, Croatia

## Background

Global tissue hypoxia portends poor outcomes. Detection and prompt interventions designed to counter the effects of inadequate tissue perfusion are paramount. The relationship between venous saturations in different venous pools remains elusive and dependent upon the patient’s hemodynamic status.

## Methods

Venous samples were drawn from the superior vena cava (S_SVC_O_2_) and pulmonary artery (MvO_2_) at three different time points (prior to operation (T0), immediately after weaning from CPB (T1) and on postoperative day 1 (T2)) from 89 consecutive cardiac surgical patients. Thermodilution cardiac indices and serum lactate measurements were obtained. Clinical outcomes were monitored.

## Results

The difference between the MvO_2_ and S_SVC_O_2_ widened over the monitored period (0.08±8.66 at T0, -1.17±7.62 at T1 and -3.12±5.88 at T2). Patients with a larger postoperative negative MvO_2_-S_SVC_O_2_ gradient had longer CPB and cross-clamp times (122±74 vs. 97±40, P=0.08; 87±48 vs. 68±26, P=0.04). This did not correlate with inferior clinical outcomes or laboratory markers of hypoperfusion.

## Conclusion

The relationship between the venous saturations between different venous pools was inconsistent over the course of the immediate postoperative period, with a tendency towards expansion of the negative MvO_2_-S_SVC_O_2_ gradient. Widening of the gradient between MvO_2_ and S_SVC_O_2_ in favor of the latter correlated with the duration of the CPB and ischemic times. Extrapolating functional cardiac performance based on S_SVC_O_2_ would be unreliable in this setting, with greater errors seen following more complex operations.

